# Case Report: Functional characterization of a missense variant in *INSR* associated with hypoketotic hypoglycemia

**DOI:** 10.3389/fped.2024.1493280

**Published:** 2024-10-17

**Authors:** Herodes Guzman, Lauren M. Mitteer, Pan Chen, Christine A. Juliana, Kara Boodhansingh, Katherine Lord, Arupa Ganguly, Diva D. De Leon

**Affiliations:** ^1^ Division of Endocrinology and Diabetes, Children’s Hospital of Philadelphia, Philadelphia, PA, United States; ^2^ Division of Human Genetics, Children’s Hospital of Philadelphia, Philadelphia, PA, United States; ^3^ Department of Pediatrics, Perelman School of Medicine at the University of Pennsylvania, Philadelphia, PA, United States; ^4^ Department of Genetics, Perelman School of Medicine at the University of Pennsylvania, Philadelphia, PA, United States

**Keywords:** insulin, beta cells, glucose, insulin receptor, hyperinsulinism

## Abstract

Hypoketotic hypoglycemia due to dysregulated insulin secretion is the most common cause of persistent hypoglycemia in children. However, this type of hypoglycemia can also result from defects in the insulin signaling pathway. Distinguishing between the two is important for informing treatment decisions. Here we describe the case of a 10-year-old female with fasting and postprandial hypoglycemia who was found to have a missense variant in the *INSR* gene, which we functionally characterized. The proband presented with fasting and postprandial hypoglycemia at age six. Diagnostic evaluation was consistent with hypoketotic hypoglycemia suspected to be due to hyperinsulinism, and she was treated with diazoxide. Whole exome sequencing identified a maternally inherited heterozygous missense variant in *INSR*. Phenotypic studies on the mother were consistent with postprandial hypoglycemia. Phosphorylated Akt and ERK1/2 levels were higher at baseline and in response to stimulation with insulin in 3T3-L1 cells expressing mutant *INSR* compared to cells expressing wild type *INSR*. Thus, herein we present a heterozygous missense variant in *INSR* (c.1151A>G, p.Asn384Ser) that results in constitutive and increased activation of the human insulin receptor, leading to both fasting and postprandial hypoglycemia.

## 1 Introduction

Congenital hyperinsulinism (HI), due to dysregulated insulin secretion, is the most common cause of persistent hypoglycemia in infants and children. It is characterized by severe and persistent hypoketotic hypoglycemia triggered by fasting and glucose or protein intake, or exercise, depending on the specific genotype. HI is diagnosed by demonstration of increased insulin secretion and actions during hypoglycemia, including detectable insulin, low free fatty acids (FFAs) and beta-hydroxybutyrate (BOHB), and a glycemic response to glucagon administration during hypoglycemia (1). More than 30 different genetic loci have been associated with HI with pathogenic variants in the genes encoding the ATP-sensitive potassium channel, *KCNJ11* and *ABCC8*, accounting for most genetically confirmed cases (2).

Dominant mutations in the insulin receptor gene (*INSR*) have also been associated with an HI phenotype, possibly due to a combination of decreased insulin degradation and differential insulin receptor function in the liver and skeletal muscle (3–10). To review, the *INSR* gene encodes for the heterotetrameric insulin receptor composed of two alpha and two beta subunits. When insulin binds to the insulin receptor at the alpha subunits, it triggers auto-phosphorylation and tyrosine kinase activity in the receptor's beta subunits (11). This in turn activates the two main insulin signaling pathways, phosphatidylinositol 3-kinase (PI3K/Akt) and Raf/Ras/MEK/MAPK (mitogen activated protein kinase or ERK). Akt and ERK1/2 (or P44/42) promote glycogen, fatty acid, and protein synthesis as well as cell growth and differentiation (12). It is important to note that genetic variants in genes downstream of the insulin receptor also have been identified as a cause of hypoketotic hypoglycemia, but in contrast to HI, beta cell function and insulin secretion is normal in these cases (13).

In this case report, we present a ten-year-old girl with fasting and postprandial hypoglycemia found to harbor a maternally inherited heterozygous missense variant in *INSR*. Through functional analysis, we show this variant to be pathogenic given constitutive and increased activity of the insulin receptor.

## 2 Case report

The proband was born full-term via spontaneous vaginal delivery with an appropriate for gestational age birth weight and no reported prenatal or delivery complications. Her growth and development were normal prior to presentation. At six years of age, she developed sudden-onset dizzy spells and was described to be pale, tachycardic and clumsy. These episodes occurred a couple hours after eating and self-resolved after a few minutes. She was initially seen by Neurology and found to have partial seizures on EEG. A brain MRI revealed no structural abnormalities nor insults. She was started on zonisamide 50 mg twice daily for approximately six weeks without observed benefit. She continued suffering from these episodes one to two times per week. The family also reported the patient was snacking every one to two hours from hunger.

During one of her dizzy spells, the patient's mother tested her blood glucose using a hand-held glucose meter, revealing hypoglycemia. The patient was subsequently seen by a local endocrinologist who recommended a diagnostic fasting study. She was fasted for a total of 18 h with plasma glucose (PG) nadir of 52 mg/dL (2.9 mmol/L) at 18 h and negative urine ketones throughout. She then consumed a small mixed meal with transient rise in PG to 141 mg/dL (7.8 mmol/L) and subsequent drop to 45 mg/dL (2.5 mmol/L) at ∼210 min after her meal. A critical sample was then obtained: confirmatory PG 32 mg/dL (1.8 mmol/L), plasma beta-hydroxybutyrate (BOHB) <0.4 mmol/L, plasma insulin 38.5 uU/mL (267.4 pmol/L) and C-peptide 4.1 ng/mL (1.4 nmol/L). Additionally, a glucagon stimulation test demonstrated a delta PG of +69 mg/dL (+3.8 mmol/L) over 20 min. Taken together, these results were suggestive of HI, and she was started on diazoxide 5 mg/kg/day. Her dosing was eventually weaned to 3.9 mg/kg/day for glucoses around 150 mg/dL (8.3 mmol/L) and she temporarily maintained euglycemia on this regimen. Afterwards, both her dizzy spells and hunger symptoms improved. A three-day EEG on this treatment revealed no abnormal brain activity and zonisamide was discontinued.

The family began noting hypoglycemia again one month after diazoxide initiation, particularly after protein-rich meals. Thus, diazoxide was increased back to 5 mg/kg/day. Despite dose escalation, she continued having hypoglycemia one to two times per week, and she was referred to our Center for further evaluation. Whole exome sequencing revealed a maternally-inherited, heterozygous, missense variant of uncertain significance in the *INSR* gene (c.1151A>G, p.Asn384Ser; Variation ID: 987803 Accession: VCV000987803.1). The mother self-reported undocumented symptoms of hypoglycemia without clear provocation. The maternal grandmother also reportedly had hypoglycemia.

Further phenotyping of the patient was completed at our Center. While on diazoxide 5 mg/kg/day, the proband fasted for 18 h maintaining PG ≥70 mg/dL (3.9 mmol/L). Although she had a normal mixed meal tolerance test (MMTT) and oral protein tolerance test (OPTT), the patient experienced hypoglycemia at +120 min on an oral glucose tolerance test [OGTT, PG 52 mg/mL (2.9 mmol/L) and insulin 6.2 uIU/mL (43.1 pmol/L)] with peak insulin level of 43.3 uIU/mL (300.7 pmol/L) reached at one hour.

The proband was weaned off diazoxide for additional diagnostic testing which was completed five days after diazoxide discontinuation. She maintained PG ≥70 mg/dL (3.9 mmol/L) for 18 h of fasting and the fasting test was terminated at 35 h with PG 44 mg/dL (2.4 mmol/L), plasma BOHB 2.2 mmol/L, and insulin 3.1 uIU/mL (21.5 pmol/L). She had a positive glucagon stimulation test with a delta PG of +36 mg/dL (+2.0 mmol/L). Repeat OGTT and OPTT were normal but MMTT provoked hypoglycemia at 5 h with PG 52 mg/dL (2.9 mmol/L). Based on these findings, the patient's diazoxide was restarted at 5 mg/kg/day divided twice daily.

On the most recent evaluation at eight years of age, the proband fasted for 18 h with PG ≥70 mg/dL (3.9 mmol/L) on a diazoxide dose of 4.6 mg/kg/day. She now reports minimal dizzy spells and no hypoglycemic episodes at home off diazoxide, which was discontinued at 9 years old.

**Figure 1:**
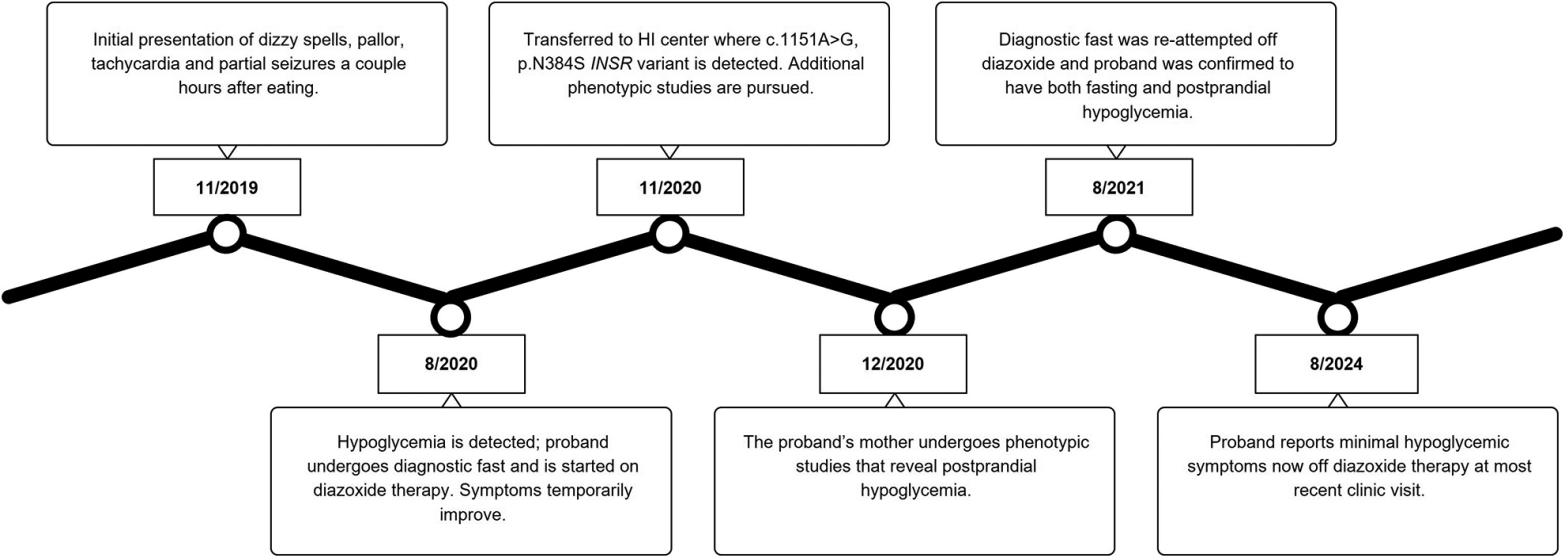
A timeline of the diagnostic course for the proband.

Given our suspicion that the patient's maternally inherited *INSR* missense variant was causing her symptoms, we completed phenotyping studies on the mother. The proband's mother was 43 years old with a body mass index (BMI) of 25.5 kg/m^2^ at the time of evaluation. She completed a 23.5-hour fasting test with labs at the end of the assessment showing a PG 74 mg/dL (4.1 mmol/L), plasma BOHB 0.3 mmol/L, FFA 0.63 mmol/L, and insulin 2.74 uIU/mL (19 pmol/L). Hypoglycemia to PG 51 mg/dL (2.8 mmol/L) occurred at +150 min into the OGTT with plasma insulin of 6.68 uIU/mL (46.4 pmol/L) and peak insulin level of 84.01 uIU/mL (583.4 pmol/L) noted at +30 min. The OPTT was not able to be completed due to scheduling restraints. Table 1 displays the results of select phenotyping assessments for the patient and her mother. Figure 1 summarizes the proband's diagnostic course.

**Table 1:** Select phenotypic findings of the proband off diazoxide and her mother.

	Time (h)	Proband				Time (h)	Proband's mother			
		PG (mg/dL)	PG (mmol/L)	Plasma BOHB (mmol/L)	Plasma insulin (uIU/mL)		PG (mg/dL)	PG (mmol/L)	Plasma BOHB (mmol/L)	Plasma insulin (uIU/mL)
DF^a^	26	62	3.4	0.8		17.5	82	4.6	0.1	
	29	60	3.3	1.0		19.5	81	4.5	0.1	
	32	62	3.4	1.5		21.5	85	4.7	0.2	
	35	44	2.4	2.2	3.1	23.5	74	4.1	0.3	2.74
OGTT	0	124	6.9		–	0	90	5.0		6.1
	0.5	–	–		–	0.5	117	6.5		84.01
	1	212	11.8		–	1	93	5.2		63.22
	1.5	188	10.4		11.5	1.5	89	4.9		46.71
	2	131	7.3		42.7	2	85	4.7		25.47
	2.5	121	6.7		6.2	2.5	51	2.8		6.68
	3	121	6.7		3.9					
OPTT^b^	−0.25	92	5.1		19.2					
	0	84	4.7		14.7					
	0.25	79	4.4		14.4					
	0.5	81	4.5		17.9					
	0.75	84	4.7		15.8					
	1	91	5.1		14.2					
	1.5	81	4.5		9.1					
	2	83	4.6		<2.0					
	2.5	83	4.6		<2.0					
	3	83	4.6		<2.0					
MMTT^b^	0	76	4.2		7.3					
	1	82	4.6		84.3					
	1.5	96	5.3		58.5					
	2	101	5.6		141.0					
	2.5	78	4.3		79.7					
	3	76	4.2		73.8					
	3.5	66	3.7		68.3					
	4	56	3.1		31.9					
	4.5	54	3.0		7.9					
	5	59	3.3		20.1					

PG, plasma glucose; BOHB, beta-hydroxybutyrate; DF, diagnostic fast; OGTT, oral glucose tolerance test; OPTT, oral protein tolerance test; MMTT, mixed meal tolerance test. Reference ranges: PG (70–99 mg/dL; 3.9–5.5 mmol/L), BOHB (≥2.0–5.0 mmol/L), FFA (≥1.5 mmol/L), insulin (≤2 uIU/mL). –, Missing data.

^a^ Final four time points of each diagnostic fast are presented.

^b^ Proband's mother did not complete these assessments.

**Figure 2:**
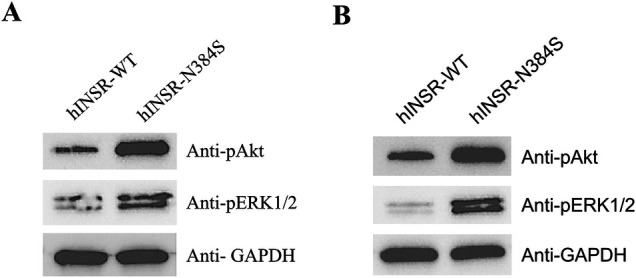
Insulin signaling is increased in hINSR-N384S expressing adipocytes independent of insulin treatment. 3T3 cells expressing either hINSR-WT or hINSR-N384S were differentiated into adipocytes and then treated ± insulin. **(A)** Western blot analysis of pAkt, pErk1/2, and GAPDH (loading control) expression in protein lysates collected from hINSR-WT and hINSR-N384S expressing adipocytes after treatment with insulin (10 nmol/L). *n* = 3 **(B)** Western blot analysis of pAkt, p44/42, and GAPDH (loading control) expression in protein lysates collected from hINSR-WT and hINSR-N384S expressing adipocytes with no insulin treatment. *n* = 3.

To assess the functional effects of the *INSR* variant on insulin signaling, we evaluated changes in phosphorylation of ERK1/2 (pErk1-Y204/Erk2-Y187) and Akt (pAkt-Thr308) at baseline and in response to stimulation with insulin in a 3T3-L1-derived adipocyte cell model expressing either the normal, control human INSR (hINSR-WT) or INSR with the identified variant (hINSR-N384S) (Supplementary Figure S1). After differentiation into adipocytes and treatment with insulin (10 nmol/L), the hINSR-N384S variant expressing cells had higher levels of both pAkt and pERK1/2 compared to the hINSR-WT control (Figure 2A). Interestingly, the increase in pAkt and pERK1/2 was also observed in the absence of insulin treatment in the hINSR-N384S variant expressing cell line compared to WT control (Figure 2B). These results indicate that the increase in insulin signaling caused by the hINSR-N384S variant is constitutive, independent of insulin binding, and effects both the mitogenic and metabolic insulin signaling pathways. Additional details on the methods for phenotyping, genotyping and functional studies can be found in Supplementary Materials.

## 3 Discussion

Variants in *INSR* have been associated with a hypoketotic hypoglycemia phenotype, but the mechanisms by which variants in this gene cause hypoglycemia have not been clearly established. Our functional analyses of the proband's c.1151A>G *INSR* missense variant demonstrate increased phosphorylation of the downstream insulin signaling mediators, Akt and P44/42, with and without insulin stimulation when compared to wild type control. This suggests that the variant results in constitutive and increased activation of the insulin receptor, which would result in both fasting and postprandial hypoglycemia and is consistent with the proband's phenotype. Not only did she exhibit fasting hypoglycemia on her diagnostic fast, but the patient also experienced postprandial hypoglycemia on her OGTT and MMTT. Furthermore, the proband's mother exhibited hypoglycemia during the OGTT even though her relatively short diagnostic fast did not capture her reported symptoms of fasting hypoglycemia.

Over 100 pathogenic variants in *INSR* have been identified to cause human disease with mutations of the alpha subunit producing a more severe phenotype (8). Most variants lead to severe insulin resistance like that seen in Donohue (OMIM #246200) and Rabson-Mendenhall syndrome (OMIM #262190). However, more recent literature reveals an association between *INSR* mutations and hyperinsulinemic hypoglycemia. It is presumed that heterozygous mutations in *INSR* lead to both insulin resistance and hypoglycemia through tissue-specific insulin receptor dysfunction. While impaired insulin receptor function at the skeletal muscle causes hyperinsulinemia and decreased peripheral glycogen formation from insulin resistance, preserved receptor function in the liver provokes hypoglycemia by suppressing glucose production in the setting of hyperinsulinemia (5, 10). Hojlund and colleagues also propose decreased insulin degradation as a mechanism of hyperinsulinemic hypoglycemia. This is evidenced by increased insulin-to-C-peptide ratios and decreased insulin clearance noted in the research team's euglycemic-hyperinsulinemic clamp studies of affected patients (3).

To date, there have been 32 cases, including the present case, in the literature reported to have heterozygous, autosomal dominant variants in *INSR* resulting in hyperinsulinemic hypoglycemia. Of these patients, seven experienced fasting hypoglycemia while 16 suffered from postprandial hypoglycemia (3–7, 9, 10, 14–16). Both fasting and post-prandial hypoglycemia have only been reported in three other cases (3, 16, 17). For five individuals, the type of hypoglycemia was not specified (3, 6). All but two variants are located on exons encoding for the beta subunit of the insulin receptor with four located on exon 20. Within exon 20, two variants result in fasting hypoglycemia while the others lead to postprandial hypoglycemia. The proband's variant is found on exon 5, encoding for part of the alpha subunit. Only one other variant is located here and has resulted in fasting or postprandial hypoglycemia within the same family (15). Thus, with the present data available, a clear genotype-phenotype correlation does not exist.

Most patients in the literature with a heterozygous pathogenic variant in *INSR* and fasting hypoglycemia developed persistent neonatal hypoglycemia shortly after birth. They were described to be small for gestational age and had mothers with gestational diabetes (10, 15). Patients often respond to diazoxide and can come off therapy around the first year of life (10). Our patient's clinical presentation was different. Her birth weight was appropriate for gestational age, and she did not have documented symptoms of hypoglycemia until 6 years of age. Her mother also did not report any concerns of gestational diabetes during this pregnancy. Of note, most reported patients with fasting hypoglycemia were not formally assessed by a diagnostic fasting study to confirm the pattern of their hypoglycemia (10, 15). Regardless, our patient's phenotype was more representative of what has been observed in the postprandial hypoglycemia cohort. These individuals presented outside of the infancy period with some individuals exhibiting asymptomatic fasting hypoglycemia on PG readings but not formal diagnostic fasting studies (3–6, 10). One notable difference is that this cohort responds well to metformin for unclear reasons. Given her appropriate response to diazoxide, we did not trial the proband on it.

Physical manifestations of insulin resistance are noted in the literature for both subtypes of hypoglycemia. Signs of insulin resistance in these individuals included acanthosis nigricans, menstrual irregularities and hirsutism (4, 6, 10). It is thought that the smaller BMIs noted in most of these individuals is a result of increased lipolysis and decreased lipogenesis in adipocytes sparked by chronic insulin resistance (5). The same idea applies *in utero* in which resistance to insulin, a potent growth factor *in utero*, results in small for gestational age status at birth (5). We did not observe any of these features in our patient nor her mother, thus highlighting the phenotypic variability observed in this patient population.

In conclusion, we present a heterozygous missense variant in the *INSR* gene (c.1151A>G, p.Asn384Ser) that results in constitutive and increased activation of the human insulin receptor, leading to both fasting and postprandial hypoglycemia without evidence of increased insulin secretion. By further elucidating the pathophysiology behind this type of hypoglycemia, we can better tailor our existing therapeutics to effectively manage the hypoglycemia affecting these patients.

## Data Availability

The original contributions presented in the study are included in the article/Supplementary Material, further inquiries can be directed to the corresponding author.
