# Supplementary material for: Case Report: Functional characterization of a missense variant in INSR associated with hypoketotic hypoglycemia
Source: Front Pediatr. 2024 Oct 17;12:1493280. doi: 10.3389/fped.2024.1493280 (PMC11524959; doi:10.3389/fped.2024.1493280)
Supplement: Supplementary file 1 [file Datasheet1.pdf]

Supplementary Materials and Methods

Phenotyping Studies

The proband underwent phenotyping studies at the Children's Hospital of Philadelphia Congenital Hyperinsulinism Center. The proband's mother was consented and enrolled into an IRB-approved phenotyping study. The protocol for each phenotyping assessment and findings are described below.

Fasting test: For the proband, the standard fasting protocol was followed and includes interval collection of plasma glucose (PG) and beta-hydroxybutyrate (BOHB). The fasting test is ended for PG <50 mg/dL (2.8 mmol/L), plasma BOHB ≥2.5 mmol/L on two consecutive draws, symptomatic hypoglycemia, or maximum duration of 36 hours. At the end of the fast, a critical sample is obtained, and a glucagon stimulation test is performed if the PG is ≤50 mg/dL (2.8 mmol/L). The mother's research fasting protocol mirrored the proband's, except for two alterations to the ending parameters: BOHB ≥2 mmol/L (instead of 2.5 mmol/L) and maximum duration of 24 hours (instead of 36 hours).

Oral glucose tolerance test (OGTT): Proband and mother received 1.75 g/kg glucose (maximum dose of 75 grams) as Fisherbrand™ Glucose Tolerance Test Beverage (Thermo Fisher Scientific, Inc., Waltham, MA) after fasting at least three hours. PG and insulin levels were collected at time 0, +30, +60, +90, +120, +150, +180, +210 and +240 minutes (proband samples at +210 and +240 minutes were not obtained).

Oral protein tolerance test (OPTT): Proband and mother received 1.5 g/kg protein (maximum dose of 60 grams) as Beneprotein® protein powder (Nestlé Health Science, Bridgewater, NJ) after fasting at least three hours. PG and insulin levels were collected at time -15, 0, +15, +30, +45, +60, +90, +120, +150 and +180 minutes (mother's samples at -15, +15 and +45 were not obtained).

Mixed Meal Tolerance Test (MMTT): Proband consumed a regular meal consisting of carbohydrates, fats, and protein after fasting at least four hours and had PG and insulin levels collected at time 0, +30, +60, +90, +120, +150 and +180 minutes.

Genotyping studies

Whole exome sequencing on the proband and both parents was performed in a commercial laboratory. Briefly, genomic DNA isolated from peripheral blood underwent fragmentation by sonication followed by enrichment of exonic regions using Agilent SureSelect^{XT} (Agilent Technologies, Inc., Santa Clara, CA) with custom content. Sequencing was performed on an Illumina HiSeq 2500 system (Illumina, Inc., San Diego, CA) and aligned using CLC-Bio Server Software, v.9.1.1 (Qiagen, Hilden, Germany). Identified variants were confirmed by targeted Sanger sequencing analysis.

Functional studies

Generation of human INSR-expressing 3T3-L1 cell lines: To evaluate downstream insulin signaling, an adipocyte cell model was generated using the pre-adipocyte 3T3-L1 cell line. First, CRISPER-Cas9 was employed to reduce endogenous mouse insulin receptor (mINSR)

expression. In pre-adipocyte 3T3-L1 cells, Cas9 (Lenti-Cas9-Blst; Addgene, Watertown, MA, catalog # 52962) expression was achieved via lentiviral infection, single cell clones selected using blasticidin (7 µg/mL), and expression confirmed in single cell clones by Western blot (Anti-Cas9, Cell Signaling Technology®, Danvers, MA, catalog # 14697) and qPCR analysis (Supplemental Fig. 1. a, b). Using Cas9 expressing 3T3-L1 cells, gRNAs designed to target mINSR were introduced via lentiviral infection by cloning into Lenti-sgRNA hygro (Addgene, Watertown, MA, catalog # 104991) to generate the Lenti-sgRNA-mINSR-hygro plasmid. Single cell clones were selected using hygromycin (125 µg/mL) and 90% reduction of mINSR expression was confirmed by Western blot (Anti-INSR; Santa Cruz Biotechnology, Inc., Santa Cruz, CA, SC-81465) and qPCR analysis (Supplemental Fig. 1. c, d) generating the 3T3-L1-mINSR-KO cell line. Finally, C-terminal FLAG-tagged hINSR-WT (wild-type) and hINSR-N384S mutant were cloned into pLenti-puro (Addgene, Watertown, MA, plasmid # 39481) to create hINSR-WT-puro and hINSR-N384S-puro plasmids. Expression was achieved through lentiviral infection of 3T3-L1-mINSR-KO cells, single cell clones were selected using puromycin (3 µg/mL), and expression was confirmed by Western blot (Anti-FLAG, Cell Signaling Technology®, Danvers, MA, catalog # 14793) and qPCR analysis (Supplemental Fig. 1. e, f) resulting in the final pre-adipocyte 3T3-L1-hINSR-WT and 3T3-L1-hINSR-N384S cell lines. These cell lines were then differentiated into mature adipocytes for assessment of downstream insulin signaling.

In-fusion cloning of lentiviral human INSR-puro plasmid and site directed mutagenesis: The lentiviral human INSR plasmid (hINSR-WT) was generated from the pLenti-puro plasmid (Addgene, Watertown, MA, catalog # 39481) and pCMV3-hINSR (NM_000208.2) (SINO Biological, Wayne, PA, catalog # HG11081-UT) using an In-Fusion HD Cloning Kit (Takara Bio USA, Inc., San Jose, CA, catalog # 639648), as per manufacture instructions. PCR fragments were separated by gel electrophoresis and purified using Takara Gel and PCR purification kit (Takara Bio USA, Inc., San Jose, CA, catalog # 740609). Transformation was completed in competent Stbl3 E. coli with the resultant plasmid. Plasmid DNA was isolated using a QIAprep® Spin Miniprep Kit (Qiagen, Tegelen, Netherlands, catalog # 27104) for colony screening. Positive clones from colony screening were verified by sequencing. Sequences for primers used in cloning and sequencing are in Supplementary Table 1.

The mutant INSR-N384S (1151 a>g, AAC>AGC(Asp>Ser)) (hINSR-N384S) was obtained by site-directed mutagenesis of the hINSR-WT template DNA using QuikChange II Site-Directed Mutagenesis Kit (Agilent Technologies, Inc., Santa Clara, CA, catalog # 200521), as per manufacturer instructions. To distinguish between ectopically expressed human INSR from endogenous murine INSR, a C-terminal FLAG-tag was added to hINSR-WT and hINSR-N384S. Plasmids were verified by sequencing.

Real-time quantitative PCR: Total RNA was extracted from cultured 3T3-L1 cells with the PureLink™ RNA Mini Kit (Invitrogen™, Thermo Fisher Scientific, Inc., Waltham, MA, catalog # 12183018A) and reverse transcribed into cDNA using High-Capacity RNA-to-cDNA Kit (Thermo Fisher Scientific, Inc., Waltham, MA, catalog # 4387406). Real-time quantitative PCR reactions were prepared in triplicate for each sample with Power SYBR™ Green PCR Master Mix (Thermo Fisher Scientific, Inc., Waltham, MA, catalog # 4367659). Expression was normalized to endogenous GAPDH.

3T3-L1 cell culture, differentiation into adipocytes, and treatment +/- insulin: 3T3-L1 cells (kindly provided by the Patrick Seale lab) were cultured in basal medium (DMEM with high glucose [Invitrogen™, Thermo Fisher Scientific, Inc., Waltham, MA, catalog # 10569010] enriched with 10% newborn calf serum [NCS; Thermo Fisher Scientific, Inc., catalog # 16010159] and 10% penicillin/streptomycin [P/S]) in a humidified incubator at 37°C, 5% CO_2. Cells were harvested with 0.25% trypsin. Single-cell clones were separated in 96-well plates by dilution and clones expressing the desired target were selected using drug resistance. Single-cell clones expressing the target gene were selected and expanded.

3T3-L1 cells were differentiated into adipocytes using modified versions previously described [1,2]. Cells were seeded in 35-mm dishes at a density of $6x10^5$ cells/dish and, at this density, cells reached confluence the next day. After 48 hours (Day 3), media was changed to Differentiation Medium I (DMEM) containing 10% NCS, 1 P/S, 0.5 mM IBMX (Sigma-Aldrich®, Inc. St. Louis, MO, catalog # I5879), 0.25 M dexamethasone (Sigma-Aldrich®, Inc. St. Louis, MO, catalog # D2915), and 1.0 mg/mL insulin, with 2 uM rosiglitazone (Cayman Chemical, Ann Arbor, MI, catalog # 1740). After 48 hours, the medium was changed to Differentiation Medium II (DMEM) containing 10% NCS, 1X P/S, and 1 µg/mL insulin (Sigma, Cat# I9278) for another 48 hours. On Day 7, the medium was changed to DMEM containing 10% NCS, 2 mM glutamine and 1 P/S (basal medium). This medium was refreshed on Days 8, 10, 12, and 13. On the evening of Day 13, we changed the media to DMEM high glucose + 0.5% BSA + P/S (without NCS serum). On the morning of Day 14, cells were treated with DMEM high glucose + 0.5% BSA + 10% P/S +/- 10 nmol/L insulin for 10 minutes in a cell culture incubator. Protein lysates were then collected for Western Blot.

Production of lentivirus and 3T3-L1 cell infection: All lentiviruses used in this study were generated in HEK293T mammalian cells via transfection and using a second-generation lentiviral system (pMG2d envelope plasmid [Addgene, Watertown, MA, catalog # 12259] and psPAX2 packaging plasmid [Addgene, Watertown, MA, catalog # 12260]). HEK293T cells were transfected with lentiviral plasmids using Lipofectamine™ 2000 Transfection Reagent (Invitrogen™, Thermo Fisher Scientific, Inc., Waltham, MA, catalog # 11668027) to produce lentivirus and media was collected at 48- and 72-hours post transfection. To harvest viral particles, the collected medium from both time points was combined and cell debris was removed via a 0.45-µm syringe filter. Cells were then infected with a lentivirus:media (1:1) mixture for 48 hours.

Western Blot analysis: Protein lysates were extracted from 3T3-L1-hINSR-WT and 3T3-L1-hINSR-N384S cells after differentiation into adipocytes +/- insulin treatment. Cells were snap frozen on dry ice, lysis buffer added, and collected by cell scrapers. Collected lysate was centrifuged twice at 14,000 rpm and 4° C for 15 minutes. The middle liquid phase was collected avoiding insoluble material and lipids. Protein concentration was determined using Pierce™ BCA Protein Assay Kit (Thermo Fisher Scientific, Inc., Waltham, MA, catalog # 23225). Gel electrophoresis was performed with 4–15% Mini-PROTEAN® TGX™ Precast Protein Gels (Bio-Rad, Hercules, CA, catalog # 4561083) and proteins were transferred to a nitrocellulose membrane (Bio-Rad, Hercules, CA, catalog # 1620146). After blocking with 5% milk, the following primary antibodies were used for analysis of insulin signaling: pAkt (Thr308; Cell

Signaling Technology®, Danvers, MA, catalog # 2965s) and P44/42 (Erk1-Y204/Erk2Y187; Cell Signaling Technology®, Danvers, MA, catalog # 5726s). GAPDH (Anti-GAPDH; Cell Signaling Technology®, Danvers, MA, catalog # 2118S) served as loading controls.

References

1. Zebisch K, Voigt V, Wabitsch M, Brandsch M. Protocol for effective differentiation of 3T3-L1 cells to adipocytes. *Anal Biochem*. 2012;425(1):88-90.
2. Brierley GV, Siddle K, Semple RK. Evaluation of anti-insulin receptor antibodies as potential novel therapies for human insulin receptoropathy using cell culture models. *Diabetologia*. 2018;61(7):1662-1675.

Figure Legends

Supplementary Figure 1. Knockdown of mINSR expression using CRISPR-Cas9 and expression of hINSR in 3T3 cells. (A) Western blot analysis of Cas9 expression in 3T3 pre-adipocyte cells and uninfected negative control cells. * indicates chosen clone. (B) qPCR results of Cas9 expression in 3T3 pre-adipocyte cells. (C) Western blot analysis of single cell clones after knock down of mINSR expression in 3T3 cells and uninfected control 3T3 cells. (D) qPCR analysis results of mINSR relative expression in 3T3 single cell clones. * indicates clone chosen for expression of hINSR. (E) Western blot analysis of hINSR-WT and hINSR-N384S expression in 3T3 cells compared to negative control. (F) qPCR results of hINSR-WT and hINSR-N384S relative expression compared to negative control.
