# Supplementary figures and images for: Case Report: Functional characterization of a missense variant in INSR associated with hypoketotic hypoglycemia
Source: Front Pediatr. 2024 Oct 17;12:1493280. doi: 10.3389/fped.2024.1493280 (PMC11524959; doi:10.3389/fped.2024.1493280)

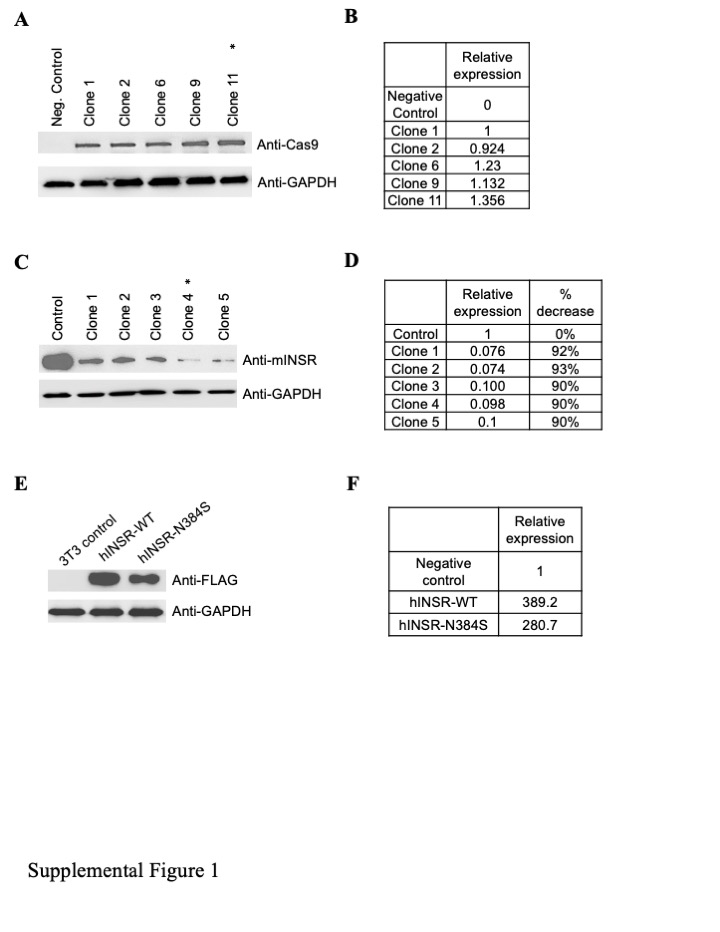

Supplement: Supplementary file 2 [file Image1.jpeg]
